# Forensic Comparison and Matching of Fingerprints: Using Quantitative Image Measures for Estimating Error Rates through Understanding and Predicting Difficulty

**DOI:** 10.1371/journal.pone.0094617

**Published:** 2014-05-02

**Authors:** Philip J. Kellman, Jennifer L. Mnookin, Gennady Erlikhman, Patrick Garrigan, Tandra Ghose, Everett Mettler, David Charlton, Itiel E. Dror

**Affiliations:** 1 Department of Psychology, University of California Los Angeles, Los Angeles, California, United States of America; 2 School of Law, University of California Los Angeles, Los Angeles, California, United States of America; 3 Department of Psychology, St. Joseph's University, Philadelphia, Pennsylvania, United States of America; 4 Department of Psychology, Technical University of Kaiserslautern, Kaiserslautern, Germany; 5 Sussex Police, Sussex, United Kingdom; 6 Institute of Cognitive Neuroscience, University College London, London, United Kingdom; National Taiwan University, Taiwan

## Abstract

Latent fingerprint examination is a complex task that, despite advances in image processing, still fundamentally depends on the visual judgments of highly trained human examiners. Fingerprints collected from crime scenes typically contain less information than fingerprints collected under controlled conditions. Specifically, they are often noisy and distorted and may contain only a portion of the total fingerprint area. Expertise in fingerprint comparison, like other forms of perceptual expertise, such as face recognition or aircraft identification, depends on perceptual learning processes that lead to the discovery of features and relations that matter in comparing prints. Relatively little is known about the perceptual processes involved in making comparisons, and even less is known about what characteristics of fingerprint pairs make particular comparisons easy or difficult. We measured expert examiner performance and judgments of difficulty and confidence on a new fingerprint database. We developed a number of quantitative measures of image characteristics and used multiple regression techniques to discover objective predictors of error as well as perceived difficulty and confidence. A number of useful predictors emerged, and these included variables related to image quality metrics, such as intensity and contrast information, as well as measures of information quantity, such as the total fingerprint area. Also included were configural features that fingerprint experts have noted, such as the presence and clarity of global features and fingerprint ridges. Within the constraints of the overall low error rates of experts, a regression model incorporating the derived predictors demonstrated reasonable success in predicting objective difficulty for print pairs, as shown both in goodness of fit measures to the original data set and in a cross validation test. The results indicate the plausibility of using objective image metrics to predict expert performance and subjective assessment of difficulty in fingerprint comparisons.

## Introduction

There has been a longstanding belief in the scientific validity of fingerprint evidence, based on the apparent permanence and uniqueness of individual fingerprints, the experience-based claims of trained fingerprint examiners, and the longstanding courtroom acceptance of this forensic technique. Yet systematic scientific study of the accuracy of latent fingerprint identification is a very recent development, still very much in progress. In the past, fingerprint identification was sometimes even claimed to be “infallible” or to have a “zero error rate” so long as the method was appropriately applied by an experienced examiner [1], [2]. High-profile cases in which errors were discovered, along with the inherent implausibility of assertions of infallibility, led to doubts about such claims of accuracy, but only in the last few years have scientific efforts to assess the strengths and limitations of fingerprint identification gained traction. The 2009 National Academy of Sciences report on forensic science [3] emphasized and spotlighted both the limits of our knowledge and the need for basic research, and since that report. The available data suggest a low level of false positive errors by experts under experimental conditions and a substantially higher rate for false negatives [4], [5]. While these data suggest that well-trained, experienced examiners are highly accurate when making positive identifications, it is also clear that errors still occur. Understanding what characteristics of print pair comparisons make errors more or less likely is thus critical to assess both the power and limits of this important forensic technique.

**Figure 1:**
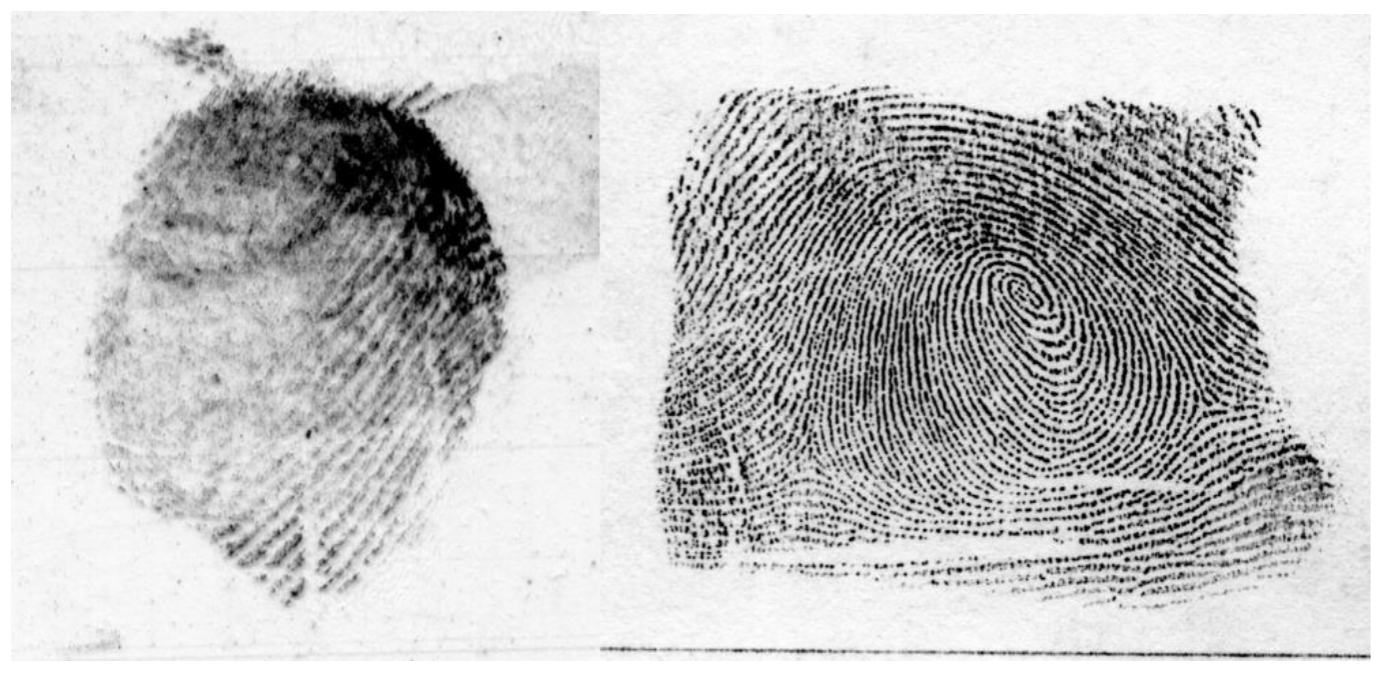
Sample fingerprint images used in the study. The image on the left is a latent print. Note large areas of the image that are smudged or missing. Contrast and ridge clarity vary greatly across the fingerprint area. These and other aspects of the image could make comparison difficult. The image on the right is a known print and is much clearer.

Fingerprint examiners can specialize and become latent or tenprint examiners or both. A latent examiner focuses on comparing “chance” fingerprints left accidentally at crime scenes or elsewhere, to possible source prints. A tenprint examiner, by contrast, compares fingerprints purposefully collected in controlled circumstances (such as at a police station) with those on file in a database. In police stations, impressions from all ten fingers are often collected on a single sheet, which is why they are called tenprints. Tenprints are also referred to as “known prints” because the identity of the source of the impression is known. In this paper, we use the term known print to refer to such prints. Latent prints have to be processed in order to be made visible, and often contain only a portion of a finger or other friction ridge area. They are often smudged, distorted, and may contain artifacts or noise due to the surface upon which they were left, or as a result of processing. By contrast, known prints are collected in controlled situations where poor impressions can be retaken, so they are typically larger, clearer, and richer in information content than latent images. Latent prints tend to be highly variable in quality, while known prints generally capture fingerprint information with high fidelity. Known prints are often acquired by law enforcement agencies using ink or a scanner. A sample latent and known print are shown in Figure 1.

Until recently, there were virtually no scientific studies of how often fingerprint examiners made errors. However, recent studies have provided helpful information for this assessment [4]–[6]. Ulery, Hicklin, Buscaglia, and Roberts [4] had 169 latent print examiners compare an independent sampling of 100 fingerprint pairs (from a set of 744), each pair consisting of one latent print and one known print. Ulrey, et al. [4] found that 7.5% of matching pairs were labeled non-matches (false negatives), while only 0.1% of non-matching pairs were labeled matches (false positives). Similar results were found by Tangen, Thompson, and McCarthy [5]: 7.88% errors for matching pairs (false negatives) and 0.68% errors for non-matching pairs (false positives). These studies took place in experimental conditions quite different from actual casework. Error rates from these studies likely do not fully reflect real-world performance [7], but they do indicate high levels of performance by experts. Studies also indicate that experts perform far better than novices at fingerprint matching tasks [5], [6].

From a research point of view, the low false positive rates among fingerprint examiners make the discovery of determinants of such errors quite difficult. High accuracy leads to little variability in performance, undermining standard statistical analyses. However, the low number of these errors should not be taken as an indication that studying them has little practical importance. A false match can lead to a false conviction, and a false exclusion can lead investigators to focus their attention on erroneous leads or to fail to convict the actual perpetrator. Furthermore, the realities and pressures in real criminal casework may substantially increase error rates, including false positives. In addition, even if these experimental error rates were established to be similar to those in actual practice, these low error rates get multiplied by a very large number of fingerprint comparisons, so the absolute quantity of real-world errors would not be *de minimus*.

Ironically, the practical importance of understanding when and why fingerprint comparison errors occur is likely to *increase* as technology advances. It is common for a latent print to be submitted to an AFIS (automated fingerprint identification system) database, where automated routines return a number of most likely potential matches. Error rates (especially of the false-positive type) may increase as databases get larger (currently some databases include tens of millions of prints). The reason for this is that as a database grows, an AFIS searching that database is increasingly likely to find close non-matches, (prints that are highly similar to the latent, but are in fact from a different individual – what are often termed “look-alikes”). Obviously, searching larger databases also increases the chances of finding a true match, but such progress can also make the task of the human examiner more demanding and, potentially, error-prone [8].

From a visual information processing perspective, it is therefore interesting and important to determine what visual characteristics of fingerprints influence the ease and accuracy of comparisons. Ultimately, it may be possible to evaluate a fingerprint comparison in terms of the quantity and quality of visual information available [9] in order to predict likely error rates in comparisons. Better understanding of objective metrics could also help determine when a print pair contains or lacks sufficient information to make an identification or exclusion, that is, to determine when an “inconclusive” assessment is warranted. These considerations motivate the present study. Its primary goals are to: (1) measure expert examiner performance, and (2) to create a predictive framework by which one could assign an appropriate level of confidence in expert decisions, derived from an objective assessment of characteristics of the pair of images involved in a particular fingerprint comparison. These two goals are interconnected: examiner performance levels (error rates) are likely to depend on the complexity and difficulty of the comparison. Specifically, as comparisons become more difficult, errors are more likely to occur. A single overall ‘error rate’ for latent fingerprint comparison would be insufficiently granular, as it would fail to recognize that some comparisons are likely far easier than others, and thus far less prone to error. Hence, the characterization and prediction of error rates must be a function of the difficulty of the comparison. Notwithstanding this relationship, no previous research on fingerprint identification has attempted to generate objective models for the assessment of fingerprint difficulty.

### Perceptual Aspects of Fingerprint Expertise

If asked to give reasons for a conclusion in a given comparison, fingerprint examiners would display significant explicit knowledge relating to certain image features, such as global configurations, ridge patterns and minutiae, as these are often explicitly tagged in comparison procedures, and they are pointed out in training of examiners. It would be a mistake, however, to infer that the processes of pattern comparison and the determinants of difficulty are therefore fully available for conscious report or explicit description. As in many other complex tasks in which learning has led to generative pattern recognition (the ability to find relevant structure in new instances) and accurate classification, much of the relevant processing is likely to be at least partly implicit [10]–[12].

Like many other tasks in which humans, with practice and experience, attain high levels of expertise, feature extraction and pattern classification in fingerprint examination involves *perceptual learning* – experience-induced changes in the way perceivers pick up information [13], [14]. With extended practice, observers undergo task-specific changes in the information selected – coming to discover new features and relationships that facilitate classification in that domain. Evidence supporting this claim comes from increased perceptual learning when these features are exaggerated during training [15].

There are also profound changes in *fluency*: What initially requires effort, sustained attention, and high cognitive load comes to be done faster, with substantial parallel processing and reduced cognitive load [12]. In turn, becoming more automatic at extracting basic information frees up resources for observers to discover even more subtle or complex structural information, e.g., [16]. This iterative cycle of discovery and automaticity followed by higher-level discovery is believed to play a significant role in attaining the impressive levels of performance humans can attain in areas such as chess, chemistry, mathematics, and air traffic control, to name just a few domains [12], [17].

While several studies have explored the influence of bias and emotional context on fingerprint matching and classification [18]–[22], there has been relatively little work investigating perceptual aspects of expertise among examiners or perceptual learning processes that lead to expertise. One exception is a study by Busey and Vanderkolk [6], in which novice and expert fingerprint examiner performance was compared on a configural processing task. Subjects were shown a small image patch from a fingerprint and, after a mask, attempted to match the image patch to a luminance- and orientation-adjusted version presented along with a distractor fingerprint image patch. Subjects did not have an opportunity to closely examine the fingerprint patches as they only appeared for one second. This presentation procedure required subjects to rely heavily on the broad patterns of ridge flow (configural information) to perform the matching task. Experts exhibited nearly perfect performance, while novices had an accuracy of 0.8. Compared to the novices, experts may have utilized fingerprint information more efficiently, focused on entirely different information, and/or more effectively filtered out irrelevant information; the study did not provide information for distinguishing among these possibilities. However, the ease of information processing of task-relevant information is a hallmark of fluency effects in perceptual learning.

Similarly, Thompson et al. [23] found that the amount of visible area in a target print was positively correlated with classification accuracy among novices. Interestingly, this relationship also depended on the source of the print (e.g., index vs. pinky), although it was unclear why one finger should hold more information than another since presented areas were constant across prints. Marcon [24] had naïve observers rate “high quality” (known prints) and “low quality” latents for distinctiveness. Performance for categorizing pairs of prints as coming from the same source or a different source was higher for high-quality and high-distinctiveness images. Together, these studies show that performance suffers when fingerprint image quality is low, but reveal little about the specific nature of the information that correlates with low or high quality.

### Fingerprint Features in the Standard Taxonomy

The first step in latent print examination is often manual preprocessing. For example, the region of the image that contains the fingerprint could be selected from the background and oriented upright. If a fingerprint is to be submitted to a database for automated comparison, key features need to be identified and labeled. Automated searches are then carried out by software that finds fingerprints on file with similar spatial relationships among the features labeled in the submitted fingerprint. This is the only part of the examination and comparison process that is automated. The software returns a list of potential matches, many of which can be quickly excluded. Some will be closer non-matches or a match, and these require further scrutiny by a human examiner.

Whether examiners are provided with potential matches via automated database searches or via investigative work, they often make their match decisions using the ACE-V approach: Analysis, Comparison, Evaluation, and Validation [25]. The examiner first looks at the latent print closely (analysis), then compares the two prints relative to each other, looking for both similarities and differences (comparison). They then evaluate those similarities and differences to arrive at a decision about whether the prints match or not. In the final step, a second examiner independently validates the conclusion. Mnookin [26] points out that there is no formalized process for any of these steps. There is no method or metric for specification of which features should be used for comparison, nor any general measure for what counts as sufficient information to make a decision. Examiners rely on their experience and training rather than formal methods or quantified rubrics at each step of the process.

**Figure 2:**
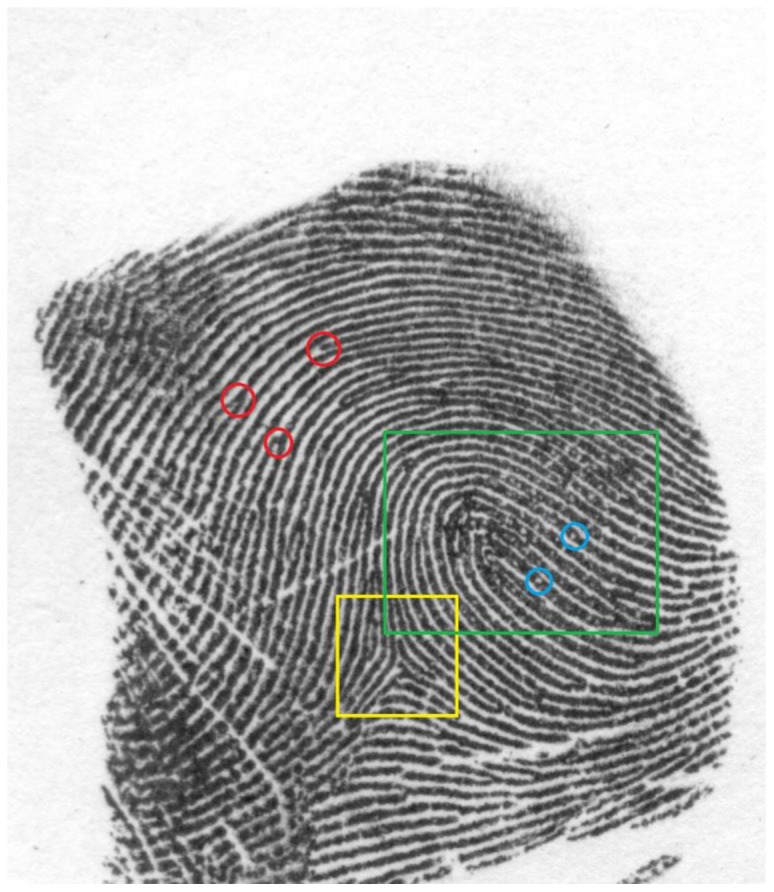
Various image features commonly identified by expert examiners. Red circles indicate *minutiae* (ridge bifurcations or endings); blue circles indicate pores (they appear as small white dots along a ridge); the yellow square indicates the delta; the green rectangle indicates the core, in this case a leftward loop.

Despite the lack of a formalized procedure, some attempts have been made to formally describe and classify the kinds of features that might be found in a fingerprint. Three types of features are commonly used to describe the information used for fingerprint comparison (for a complete discussion, see [27]). Level I features are global descriptors of ridge flow easily seen with the naked eye. These include patterns in the central region (the “core”) of the fingerprint. Cores can be classified into a limited number of typical patterns such as left- and rightward loops, whorls, tented-arches and arches. Deltas are another Level I feature that are triangular patterns of ridge flow that often occur on the sides of loops and whorls. A leftward loop and a delta are indicated by the yellow and green boxes respectively in Figure 2. Level I features are too common to be sufficient for identification, but they can be used for exclusion purposes as well as to guide inspection of the more detailed Level II and Level III features.

Level II features include *minutiae* such as ridge bifurcations and ridge endings. Level II features are found where fingerprint ridges and valleys split or end. *Minutiae* are highlighted in red circles in Figure 2. The power of fingerprints for identification purposes is largely due to the high variability in the existence and relative positions of these features across fingers and individuals. Scarring, which occurs naturally with age and wear, can also add unique ridge patterns to a fingerprint. However, while scars can be used to compare the fingerprint found at a crime scene to that of a suspect in custody, they may not always exist in fingerprints on file that can be old and therefore predate the markings.

Level III features are the smallest fingerprint features used by some examiners for comparison. These include the positions of sweat pores and ridge thickness. Pores are indicated in light blue circles in Figure 2. The visibility of Level III features depends on the quality of the prints and examiners do not uniformly make use of them for comparison purposes.

### Predictor Variables

What properties of the images in fingerprint pairs are most important and informative in comparing fingerprints? What visual qualities of individual prints or of print pairs make accurate matching performance more or less likely? Although we relied on regression methods to provide answers to this question, it was important to develop as inputs to the regression analyses a wide variety of possible image characteristics that could be relevant. To generate such factors, we were guided by vision science, intuition, insights from fingerprint examiners, and prior work on image processing of fingerprints [27], as well as the standard taxonomy of levels of pattern information in fingerprints (described above). Some variables intuitively seem likely to relate to the sheer quantity of available information; for example, having greater print area available for comparison might make comparisons more accurate. However, this might well be oversimplified; quality of information might matter as much or more than total print size. We created several image quality metrics that appear sensitive to smudging, missing regions, poor contrast, etc. These metrics were computed in an automated fashion on the fingerprint images themselves, and were designed to relate in a variety of ways to the presence or absence of visual information that examiners use, and could therefore function as independent variables that are predictive of examiner performance.

We hypothesized that difficulty would be a function both of the characteristics of the individual prints (the latent and the potential match) and also of the characteristics of the *pair*. Because known prints are obtained under relatively standardized conditions, they are subject to significantly less variability than latent prints obtained from crime scenes. Accordingly, we expected that more of the variability in visual information quality affecting fingerprint comparisons would be determined by characteristics of latent prints. An especially poor quality latent might be more difficult to assess than a higher quality one, all else being equal. However, we also believed that pair difficulty would be a function of interaction effects between the latent and the known, not simply a function of the information quality and quality of each independently. We therefore developed quantitative measures involving both individual prints and print *pairs*.

A general description and motivation for the image features we selected or developed is provided below. Except where noted, we assessed each predictor variable for both the latent print and the known print. For many variables, we also derived a variable that expressed an interaction or relationship of the values of a variable for the latent and known print combined (such as the ratio of latent print area to the known print area, or the Euclidean sum of contrast variability for the latent and known print combined). For details about the procedures used to derive the measures we used, please see the supplementary materials.

#### Total Area

This variable was defined as the number of pixels in the fingerprint after the fingerprint was segmented from the background. Although machine vision algorithms exist that could have been used for determining the region of usable print image, those algorithms we examined were not as good as human segmentation, and different human observers in pilot work produced strong agreement. Accordingly, we segmented fingerprints from their surrounds by having human observers designate their boundaries (see supplementary materials for details). In general, we expected that larger areas, especially of latent prints, would provide more information for making comparisons.

#### Area Ratio

To relate the relative area of a latent to a potentially matching known print, we divided the area of the latent fingerprint by the area of the known print. Typically the known print, obtained under controlled conditions, presents a more complete image. Thus, *Area Ratio* relates to the proportion of known print information potentially available in the latent print. However, for non-matching prints, the area of the latent may be larger than that of the known print because of differing finger sizes. Occasionally, even for a matching latent and known print, the latent could be larger than the known print due to smearing. The ratio was therefore not strictly in the range [0,1] and cannot be considered a true proportion.

#### Image Intensity

We measured the mean and standard deviation of pixel intensity taking into account all of the pixels in each fingerprint image (with intensities scaled in the range of [0,255]). The mean intensity and standard deviation of intensity provide two related but different measures, sensitive to different image characteristics. Very dark images (low mean intensity) might indicate the presence of large smudges that produce large, dark areas. Low standard deviation in intensity would make ridges (transitions from light to dark) difficult to detect.

#### Block Intensity

The image was divided into 50×50 pixel regions and the average pixel intensity was computed within each region. The mean of the block intensities is the same as the overall mean *Image Intensity*. The standard deviation of these regional averages (*standard deviation of block intensity*), however, can provide additional information about variability in image intensity across the image. Low variability is indicative of many similar areas across the image, but does not provide information about whether those regions have low or high contrast (i.e., an all black image and an image with 50% white and 50% black pixels, evenly distributed across the image would have low *Block Intensity* variability). When pixel intensities are not uniformly distributed across the image, variability of block intensity is high (i.e., some regions of the image are darker than others). For latent images, this may indicate the presence of a smudge or worse contact (lighter impression) in some regions of the image.

#### Deviation from Expected Average Intensity (DEAI)

Intensity, as coded above, may be a useful predictor variable, but both intuition and pilot work led us to believe that it might not capture some significant aspects of intensity variations. We therefore developed a separate intensity measure – deviation from expected average intensity. In an ideal fingerprint image, one might expect approximately half of the pixels to be white (valleys) and half to be black (ridges). The expected mean intensity would therefore be half of the range, or 127.5 (with the brightest pixel normalized to 255 and the darkest to 0). The absolute deviation of the observed average from the expected average was computed using the following formula: 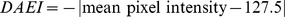



Using absolute value here ensures that deviations from the midpoint of the intensity range in either direction are scored as equivalent; the negative sign ensures that the measure increases as the mean pixel intensity approaches 127.5 (large deviations produce a large negative value of the measure). While ridges (black regions), on average, are thicker than valleys (white regions), making the average intensity slightly lower than 127.5, the difference was relatively small and was ignored.

#### Contrast

Michelson contrast was computed for each segmented fingerprint. Michelson contrast is defined as:
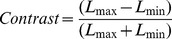



This contrast measure produces a value between 0 (least contrast) and 1 (most) by dividing the difference of maximum and minimum intensity values by their sum. Michelson contrast is typically calculated from luminance values. In our images, we calculate Michelson contrast from pixel intensity values, which is appropriate given that fingerprint images may be displayed on a variety of monitors with different Gamma corrections.

#### Block Contrast

The preceding measure obtained the Michelson contrast for an entire image. We also computed contrast for smaller image regions – block contrast – by segmenting the entire image into 50×50 pixel regions. *Block Contrast* is defined as the mean across the blocks. To illustrate the difference between overall contrast and block contrast, the Michelson contrast of an entire image containing all gray pixels except for one white and one black pixel, would be 1. *Block Contrast*, however, would be very low, since most regions of the image would have 0 contrast. If black and white pixels were distributed more evenly across the image such that they appeared in each block, then *Block Contrast* would be high. High values of the measure may indicate the presence of clear ridges and valleys in many areas of the fingerprint. A separate but related predictor was the *standard deviation of block contrast* across blocks. Small standard deviation values could indicate high information content throughout the image (*Block Contrast* close to 1 everywhere) or that the image was uniformly smudged (*Block Contrast* close to 0 everywhere).

#### Ridge Reliability

Orientation-sensitive filters were used to detect edges in the fingerprint image. The relative responses of these filters were then used to identify “high reliability” regions where ridge orientation was uniquely specified (see supplementary materials for details). The proportion of high reliability regions was computed, resulting in an overall reliability score for each print. Ridge Reliability ranged between 0 and 1, with larger values indicating a greater proportion of print area with well-defined ridge orientation. An additional, relational predictor was computed by taking the Euclidean sum of the *Ridge Reliability* for the latent and known print (*Ridge Reliability Sum*). Large values of this measure indicate a high proportion of regions with well-defined ridge orientation in both the latent and known prints.

#### Visibility of Cores and Deltas

Earlier we described global configurations – *Cores* and *Deltas* – that provide Level I information to fingerprint examiners. The fact that ridge flow in fingerprints tends to follow a circular pattern dictates that there will be some global core (a whorl, loop, or arch) at or near the center of each print. Likewise the transition from a core, especially loops and whorls, to the circular ridge flow tends to give rise to deltas (see Figure 2). As there will be only one core and at most a small number of deltas in any print, these serve as important reference points in making comparisons [27]. Unlike all of the other variables we used, which could take on a continuous range of values, *Cores* and *Deltas* are binary (either present or not).

### Relations Among Basic Predictors

To remove effects on regression coefficients of differing scales of various predictors, we standardized all continuous metrics by subtracting the mean and dividing by the standard deviation. Standardization made some measures that were strictly non-negative (like *Standard Deviation of Intensity*) take on negative values. As is often recommended in using regression methods [28], we also examined the variables for collinearity and found that several predictors were highly correlated. For example, the mean and standard deviation *Intensity* measures were correlated (Pearson's r = −0.77 for latents and −0.44 for known prints). High correlation among predictors is an undesirable feature for regression models [28] because it makes it harder to assess the individual effect of those predictors. If two predictors had a correlation of greater than 0.5, we removed one of them. After removal, the variance inflation factor, a measure of collinearity, for all continuous metrics was less than 5, indicating that collinearity was sufficiently reduced [28]–[30].

In addition, we included two-way interactions between all predictors that applied to both a latent and known print. For example, in addition to the *Standard Deviation of Block Contrast* for the latent and known print, we included the interaction between the two terms. In addition to *Area Ratio* and *Ridge Reliability Sum*, these interactions are *relational* predictors that encode something about the relative quality of information in a latent and known print.

### Overview of the Experiment

We developed a database of fingerprint images, both of the known prints and their corresponding latents, and computed a variety of metrics that we hypothesized would relate to image quality and information content. Our primary focus was accuracy, but we also measured response times and asked experts to provide subjective judgments of difficulty and confidence for each print pair. We tested expert fingerprint examiners in a task requiring a forced-choice judgment of whether two prints matched. As will be described below, the task approximated what examiners do in their real-life work in some ways but differed in others. For example, in the study reported here, images appeared on a computer monitor; examiners were limited in comparison time for each pair; and judgments were constrained to indicating that a pair of prints came from the same source or different sources, i.e., “inconclusive” was not a permitted response for difficult comparisons. These features of our design were chosen so that we could collect important data, including best-guess match determinations for difficult comparisons, and to permit us to obtain enough data to allow us to explore the set of image characteristics that might predict difficulty. We fit a regression model to measure how various image characteristics predict performance. To foreshadow some of the results, we found that a subset of image features such as measures of the reliability of ridge orientation information, the ratio of the visible area of the latent to the known print, and measures of contrast and intensity information were predictive of performance. The model accurately identified print pairs that had low accuracies, suggesting that it can be used as a valid tool for identifying potentially difficult comparisons and that in general, it may be feasible to use these methods to predict error rates for print pairs, as a function of comparison difficulty, with reasonable accuracy.

## Method

### Ethics Statement

This study was performed in accordance with the guidelines of the Declaration of Helsinki. All experts provided written, informed consent after the general purpose of study was explained and were fully aware of the purpose and procedure of the study. Participation was voluntary. The study was approved by the institutional review board of the University of California, Los Angeles.

### Participants

Fifty-six fingerprint examiners (18 male, 35 female, three not reported) participated in the study. Forty participants self-reported as latent print examiners, three as known print examiners, ten as both, and three did not report. Years of experience were reported between the range of 1 and 25 years (Latent: Mean = 9.54, SD = 6.97; Ten-Print: Mean = 10.45, SD = 8.07). Twenty-seven participants reported being IAI certified. 32 reported that their labs were accredited.

Participants were either directly recruited at the 2011 IAI (International Association for Identification) Educational Conference or via a flyer sent out in advance of the conference. As incentive, all participants were entered into a raffle to win an iPad 2. All participants signed informed consent forms prior to participating. As indicated above, some limited demographic information was collected, but it was stored separately from individual participant IDs such that the two could not be linked.

### Apparatus

All stimuli were displayed on laptop computers with 17-inch monitors at a resolution of 1024×768 pixels. Stimuli were presented using a program accessed online; data were stored on the website's server.

### Stimuli

Fingerprints were collected from 103 individuals. Each individual first used a single finger to produce a clear, known print using ink as is often done in police stations. Then, using the same finger, they touched a number of surfaces in a variety of ways (with varying pressure, smudges, etc.), to create a range of latent fingerprint marks that reflect those found in a crime scene. Professional fingerprint examiners who participated in the study reported that these prints were similar to those that they encounter in their everyday casework). The latent fingerprints were lifted using powder and were scanned at 500 dpi using the FISH system. Image dimensions ranged from 826 pixels in height to 1845 pixels and from 745 pixels in width to 1825 pixels. The latent prints varied in clarity, contrast, and size. For each individual who contributed to the database, we collected a total of six prints – one known print and five matching latent prints. Across individuals we varied the fingers used. Each scanned fingerprint was oriented vertically and approximately centered. Some individuals contributed multiple sets of prints from different fingers.

To create the non-matching pair of prints, we did not want to randomly select a known and a latent, as such pairs would often be too obviously different. This would make the “non-match” decisions nearly uniformly easy, and would also, by default, indicate which were the “matching” pairs. Therefore, we obtained similar, but non-matching, known prints by submitting each latent print to an AFIS search process. An expert selected from the AFIS candidate list what he deemed to be the most similar non-matching print. That enabled us to produce non-matching pairs with a relatively high degree of similarity. The final database consisted of 1,133 fingerprint images – five latent prints from 103 fingers (515), 103 known prints that matched (103), and another 515 known prints, to provide a potential non-match for each of the latents. Since we used an AFIS database from a different country from where we collected the known prints, it was highly unlikely that an actual match would be presented by the AFIS database search as a candidate. Furthermore, the expert who selected the most similar print from the AFIS candidate list verified for each comparison that this was a similar print, but not an actual match.

Of the 1,133 fingerprint images, 200 latent and known print pairs were selected and used for the study; half were a match and half were a close non-match. Individual print metrics were computed for each image or image pair (see below) and prints were selected to (approximately) uniformly sample each feature space. Known prints were sampled without replacement, but multiple latent prints from the same finger were occasionally selected since each latent could be paired with a different known print image (the match or a close non-match). Print pairs were then grouped into batches of 20, each containing ten matches and ten non-matches. Latent prints from the same finger did not appear within the same batch.

### Design

A group of experts made match/non-match judgments and provided confidence and difficulty ratings on a subset of 200 print pairs selected from a database of over a thousand fingerprint images. Two fingerprint images that were either from the same finger (match) or from two different fingers (non-match) were presented side-by-side. Images were presented on computer screens and were oriented upright. Examiners had a maximum of three minutes to evaluate each pair of images. Performance was recorded for each print-pair tested, and a model was fit predicting performance based on the set of image features computed for each image in the database.

### Procedure

Participants were tested in a large room, seated at desks with individual laptop computers. Before data collection began, each participant was asked to sign a consent form, and then given written instructions detailing how the stimuli would be presented and the judgments they would be required to make. Participants were told that they would be asked to compare latent-known print pairs and determine whether they were matches or non-matches (without the option to choose “inconclusive” as a response). Participants were also told that they would be asked for confidence and difficulty ratings for each of their judgments. The instructions emphasized that this procedure was not intended to replicate real-world conditions and that participants should simply try to maximize accuracy. Participants were also instructed to refrain from using any fingerprint examiner tools not provided by the experimenter, such as a compass.

When the experimental program was initiated, participants were asked to report their age, gender, years of experience, specialization, IAI certification, lab accreditation, and lab affiliation. Reporting this information was optional.

Next, the experiment began. On each trial, two fingerprints were presented side-by-side. The latent print was always on the left. A button in the top-left corner of each image window allowed participants to zoom in on each image individually. Fingerprint image size was constrained within the bounds of each window, so that each print was always viewed through an aperture of 460 pixels by 530 pixels. The initial presentation of the images had them scaled to fit entirely in this window. A single level of zoom allowed participants to magnify the image. Participants could also translate each image independently within its window (both when the image was zoomed or unzoomed) either by dragging it with mouse or by using arrow buttons in the top-left corner of each image window. No other image manipulation features were available.

Participants made a match/non-match judgment by clicking a button at the bottom of the screen. Specifically, participants were asked: “Do these prints come from the same source or a different source?” Participants then made difficulty and confidence ratings by clicking on a Likert scale. The participants were asked: “How difficult is the comparison?” and “How confident are you in your decision?” On the Likert scales, “1” corresponded to least difficult/least confident and “6” corresponded to most difficult/most confident. Once all responses were recorded, an additional button appeared allowing the participant to advance to the next trial. Supplementary Figure S1 shows a sample screenshot of the experiment.

Participants had three minutes to complete each trial. A message was given after two and a half minutes warning that the trial would end in 30 seconds. If the full three minutes elapsed without a decision, that trial was ended, and the participant moved on to the next trial. After presentation of a set of 20 print pairs, participants were given a short break and asked if they wanted to complete another set of 20 comparisons.

Each set of 20 print pairs contained ten match and ten non-match comparisons, though examiners were not provided with this information. The order in which print pairs were presented within a set was randomized across subjects. The sets were presented in a pseudo-random order so that approximately ten participants completed each set. Although the number of trials completed by individual participants varied based on their availability and willingness to do more comparisons, most participants completed two sets of prints (40 print pairs).

## Results

### Data Preprocessing

If the participant made a match/non-match judgment, but time expired before they could make difficulty or confidence ratings, the data were retained. There were thirteen such trials. If only difficulty and confidence ratings were provided, but a comparison judgment was not made before time expired, the trial was excluded from the analyses. Twenty such trials were excluded from the total of 2,312 comparisons (fewer than 1%). For one subject, time expired on eight of the trials they completed. There was no consistency in which print pairs had time expire – for two of those pairs, time expired for two subjects, for the rest, time expired for only one subject.

### Descriptive Statistics

Responses were aggregated across participants and prints. Overall accuracy (percent of correctly classified latent-known print pairs, averaged across subjects) was 91% (range: 8.3–100%, SD 17%). Overall accuracy was 86% for “match” trials (14% false negatives) and 97% for “non-match” trials (3% false positives). Of the 2,292 comparisons, there were 200 errors, resulting in an overall error rate of 9.6%. There was some variability in performance among experts (range: 79–100%, SD 5%).

**Figure 3:**
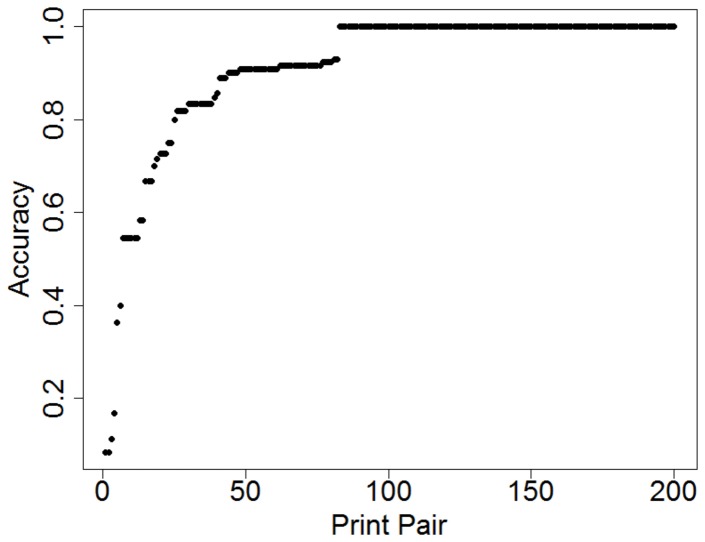
Sorted average accuracy for each print pair. Print pairs are numbered along the x-axis from 1–200 in order of increasing accuracy.

Across all participants, 118 of the 200 print pairs produced 100% accuracy. Mean difficulty and confidence ratings for these pairs were 2.62 and 5.23 respectively, compared to ratings of 4.06 and 4.15 for prints that were misclassified by at least one participant. Of the118 pairs that produced no errors, 72 were non-matches and 46 were matches. The lowest accuracy, 8.3% (1/12), corresponded to false negatives for a “match” print-pair. Average accuracy for each print pair is shown in Figure 3 sorted by increasing accuracy.

### Correlations Among Dependent Measures

We measured the correlations among the three dependent measures. There was a strong negative correlation between average difficulty and confidence ratings (*r*(198) = −0.91, *p*<0.001) and weaker correlations between average accuracy and confidence (*r*(198) = 0.52, *p*<0.001), and between average accuracy and difficulty (*r*(198) = −0.50, *p*<0.001). These results suggest that experts' confidence in their judgments is well matched to their perceived difficulty of the judgments, and further that both expert's perceived confidence and difficulty are predictive of performance. There was also a strong positive correlation between response time (RT) and difficulty (*r*(198) = 0.71, *p*<0.001) and a negative correlation between response time and confidence (*r*(198) = −0.59, *p*<0.001). Accuracy was highest and RT lowest for prints that were rated least difficult. Accuracy decreased and RT increased as print difficulty ratings increased. Altogether, these results suggest that experts faced with difficult print comparisons tend to have lower confidence in their judgments, and take more time to ultimately make a match/non match decision. Excluding the 118 prints with 100% accuracy, the correlations between accuracy and confidence and between accuracy and difficulty were qualitatively weaker, but the difference did not reach significance. The full set of correlations is shown in Table 1.

**Table 1:** Correlations between dependent measures.

		Accuracy	Confidence	Difficulty
All Print Pairs	Confidence	0.52***		
	Difficulty	−0.50***	−0.91***	
	Response Time	−0.48***	−0.59***	0.71***
Print Pairs with Accuracy <100%	Confidence	0.36**		
	Difficulty	−0.32**	−0.89***	
	Response Time	−0.22*	−0.34**	0.45***

Note. *** *p*<0.001, ** *p*<0.01, *p<0.5.

### Regression Analyses

We fit a crossed, logistic regression model in which print pair performance (1 =  accurate; 0 =  inaccurate) was crossed with expert and print identity. This is a type of mixed-effects model and is appropriate for analyzing these data for several reasons [31], [32]. First, not every subject evaluated every print pair. A mixed-effects approach enables the examination of both the predictor variables and the random effects due to inter-subject differences (i.e., differences between expert performance and differences between evaluations of the same print pair by multiple experts). Second, a mixed-effects approach allows one to model individual item differences by fitting data from individual trials instead of aggregating across all presentations of an item [33], [34]. Differing levels of expertise and experience, as well as differences in comparison strategy and decision thresholds, could give rise to variability in participant performance independent of the fingerprint features. Variability across items could occur if some comparisons were easier than others irrespective of differences in measured image features. Including these sources of variability in the model allows us to test whether print comparisons and experts differed from one another, instead of assuming they were all equivalent, and simply averaging across participants and items. Data were fit using the “arm” [35] and the “lme4” [36] R packages for R version 2.15.2.

For each of *i* print-pair comparisons (items) and *j* experts (subjects), we define *y_i,j_* as 
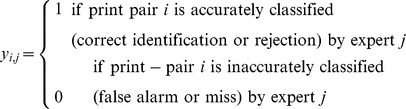
where X*_i,j_* is a vector describing the features measured on a print pair, *β* is a vector of coefficients (the fixed effects; one coefficient for each feature), expertID*_j_* is the expert-specific random effect, which allows the intercepts to vary across experts, and printID*_i_* is the item-specific random effect. expertID and printID were normally distributed.

The regression equation can be rewritten and expanded as: 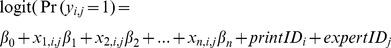
(2)where *n* is the number of predictors. In this form, it can be seen that printID and expertID can be grouped with *β_0_* as intercept terms. Because printID and expertID are vectors, the equation reflects that each combination of print and expert has its own intercept term. It is this combined term (*β_0_* + printID*_i_* + expertID*_j_*) that varies across experts and items. Multi-level modeling allows one to capture possible differences between individual subjects or test items without fitting a separate regression equation for each item (by applying a distribution over the terms that vary, in this case printID and expertID; see [37]).

Individual differences among experts may arise due to differences in experience, training, and other factors. These could manifest themselves as different baselines of performance, or intercept terms in the model. All else being equal, one expert might do better with the exact same print pair than another expert. This variability is captured by the expertID term in the model. It is also possible to model item-specific (in this case, print-pair-specific) effects; these are represented by printID. PrintID captures differences in print comparison difficulty inherent to individual print pairs and not related to the features used to predict print pair accuracy. In constructing a model, it is assumed that the error terms are uncorrelated; however, it is possible that print pair errors are correlated across participants. Inclusion of the item-specific term captures this potential non-independence (Baayen et al., 2008). A likelihood ratio test showed that the model with the predictors fit the data better than a null model with only the random effects terms (χ^2^(17) = 53.27, *p*<0.001).

Comparing a model that included the random expert effect (expertID) to one that did not, we found that the Akaike Information Criterion (AIC) was slightly smaller for the model that included the effect, but the Bayes Information Criterion (BIC) was smaller for a model that did not. Both of these measures are information-theoretic metrics of goodness-of-fit that take into account overfitting of the data with excess parameters. Qualitatively, a more parsimonious model that fit the data almost as well would have a smaller AIC and BIC [38], [39]. The fact that the criteria move in opposite directions when the model includes expertID suggests that any differences between the models should be treated with caution [38]. A likelihood ratio test comparing the two models was significant (χ^2^(1) = 4.79, *p*<0.05). ExpertID terms varied from between −0.52±0.69 to 0.44±0.77. All values of expertID were within two standard errors of zero. In terms of Equation 2, this means that *β_0_* + expertID was not reliably different from *β_0_*. Based on these analyses, we felt justified in averaging across experts and ignoring between-expert differences in all subsequent modeling steps by removing the expertID term. This same analysis could not justify excluding the print-pair specific term, printID, and so it was retained in the model.

We simplified the model further by removing predictors (fixed effects) based on minimization of the AIC [40]. A likelihood ratio test revealed no statistically significant difference between a model that included all of the predictors and the reduced model (χ^2^(11) = 9.55, *p*>0.05), indicating that the removal of predictors increased parsimony without significantly impacting predictive ability.

This analysis included all print pairs used in the study. This was done because the goal of the study was to create a model of difficulty for novel comparisons for which ground truth regarding whether or not a print pair shares a common source is unavailable. A separate analysis using only matching pairs showed highly similar results, including all of the predictors that proved to be reliable in the main analysis.

### The Accuracy Model

The model obtained for accuracy was: 
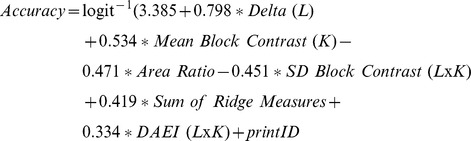



Where L and K indicate whether the predictor applies to a latent or known print image respectively, and LxK indicates predictors that apply to print pairs. printID is the item-specific, random effect. The parameters of the fitted model are shown in Table 2. All predictors were significant (Wald's *z*, *p*s<0.05), except for Delta (L) and DEAI (LxK) which were marginally significant (*p* = 0.054 and *p* = 0.053 respectively). It should be noted that there is some disagreement on how to calculate *p*-values for the Wald statistic in unbalanced, mixed-effects data due to difficulty in determining the appropriate degrees of freedom and they should therefore be interpreted with caution [32], [41], [42].

**Table 2:** Predictors for accuracy model.

Fixed Effects	Coefficient Estimates	Standard Error	z
Intercept	3.385	0.197	17.167***
Delta (L)	0.798	0.415	1.923
Mean Block Contrast (K)	0.534	0.164	3.268**
Area Ratio	−0.471	0.156	−3.010**
SD Block Contrast (LxK)	−0.451	0.128	−3.530***
Ridge Sum	0.419	0.154	2.715**
DEAI (LxK)	0.334	0.173	1.938
Random Effects	Variance		
printID	2.154		

Note: *** *p*<0.001, ** *p*<0.01. p-values are reported here, but should be interpreted with caution. They were not used for model selection. Estimates are arranged by coefficient magnitude in descending order (see text). L – latent, K – known print, LxK – interaction.

To get a more intuitive notion of model performance, we used the predicted proportions from the logistic regression as estimates of average performance across experts. The resulting fit was very good (*R* ^2^_adj_ = 0.91). We also computed the root mean squared error (RMSE) by taking the sum of the squared differences between predicted and observed values. Values closer to 0 indicated better performance. The error for the fitted model (RMSE_model_ = 0.06) was lower than for a null model that only included the printID random effect (RMSE_null_ = 0.18).

### Validation of the Regression Model for Accuracy

The dataset was split into training and testing sets. The training set contained 180 (90%) of the print pairs (2063 individual observations), and the testing set contained the remaining 20 print-pairs (10%, 229 observations). The testing set print pairs were a representative sample of the overall dataset, containing 12 pairs with perfect accuracy and 8 pairs with less-than-perfect accuracy. This was important in order to ensure that the training set did not have too few pairs with low accuracies (there were only 24 pairs with average accuracies below 80%). We replicated the model selection procedure for data only from the training set. The same predictors were selected with comparable coefficients, except for Delta (L) which was replaced with Core (L). For both the full and training datasets, the coefficients for these two predictors, Delta (L) and Core (L), were not significantly different from zero and were within two standard deviations of zero. Nevertheless, they could not be excluded based on the selection procedure described above. The fit of the model to the training set was comparable to the fit of the model to the full set (*R* ^2^_adj_ = 0.89, RMSE_train_ = 0.07).

**Figure 4:**
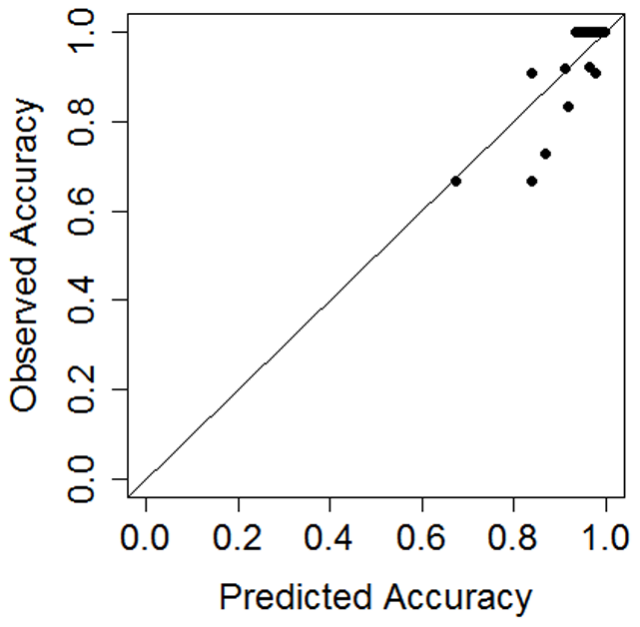
Model performance. Model predictions of average accuracy for 20 test print pairs plotted against observed average accuracy.

We used this regression model fitted to the training set to predict accuracy for the withheld testing set of 20 print pairs. The percentage of variance explained was worse for the testing set than for the training set, suggesting some amount of overfitting (R^2^_adj_ = 0.64). The error, however, was comparable between the training and testing sets (RMSE_test_ = 0.07). The model's predictions are shown in Figure 4.

As a secondary assessment of model performance, we used the model to predict whether at least one expert made an error on a print pair. We divided the set of print pairs into two classes: those that had 100% accuracy (perfect pairs) and those that had less than 100% accuracy (non-perfect pairs). A naïve classification strategy not based on the model and that assumes no errors are ever made would have a classification accuracy of 107/180 or 59%. Using the model fitted to the training set, we parametrically varied a classification threshold such that print pairs with a predicted accuracy greater than or equal to that threshold were classified as perfect pairs and those below that predicted accuracy were classified as non-perfect pairs. A threshold setting of 94% resulted in the best classification performance of 164/180 or 91% correctly labeled pairs.

The classification procedure described above was repeated for predictions generated for each left out (testing) pair using the threshold optimized on the training set. 75% (15/20) of the pairs were correctly classified as either having perfect (9/15) or non-perfect accuracies (6/15). The classifier was slightly better at correctly identifying print pairs that had at least one error than those that were perfect: 3 perfect prints were misclassified as having an error and 2 non-perfect pairs were misclassified as perfect.

### Difficulty Ratings

Difficulty ratings showed a reliable negative correlation with accuracy (see Descriptive Statistics, above), indicating that experts had reasonable metacognitive awareness (i.e., print pairs that were thought to be difficult tended to have lower accuracy across experts). Accuracy for prints with an average difficulty rating greater than 3 (on a scale of 1 to 6) was 84% compared to 91% for all prints. We compared the fitted model from the previous section to one that also included difficulty rating as a predictor. The resulting model had significantly better goodness of fit than the model described in the preceding section that did not include difficulty rating as a predictor (χ^2^(1) = 81.1, *p*<0.001, RMSE_model+difficulty_ = 0.05, R^2^_adj_ = 0.95).

We added difficulty rating as a predictor for the regression model applied to the training set described above. Predictive performance on the testing set was worse (decreased *R* ^2^) than when the difficulty rating was not included. However, classifier performance on the testing set was slightly improved, with 85% (17/20) of the pairs classified correctly. One perfect print was misclassified as non-perfect, and two non-perfect prints were misclassified as perfect. The discrepancy between the relatively worse regression fit and the improvement in classifier performance is due to two non-perfect print pairs that had a predicted accuracy that was much lower than their true accuracy. These were classified correctly as non-perfect, but contributed significantly to the error of the regression fit.

The inclusion of difficulty ratings in applications of this model must be made with caution. All other measures capture objective features of the fingerprint image, while difficulty ratings are subjective and therefore may vary across individuals and rely on the good faith of the raters. Therefore, while difficulty rating may be informative to include, in subsequent models we opt to exclusively deal with objective factors. We return to this point in the discussion.

### Regression Analyses of Other Dependent Measures

Difficulty ratings, confidence ratings, and response times were reliably correlated with accuracy and so ought to also depend on print pair information content. If similar features are predictors for many measures, then they are likely capturing something important about the fingerprint images. Here, we fit models of the other dependent measures to the training dataset as a further validation step: the importance of particular image features as valid predictors of accuracy is bolstered if those same features are shared in models of other dependent measures.

Unlike accuracy, response time varied greatly across experts, with some experts taking much longer times on comparisons that other experts evaluated fairly quickly. There are several possible reasons for this variability. Less experienced examiners may take longer to come to the same conclusion than a seasoned examiner (a perceptual fluency that comes with expertise; see [12]). Some subjects may have completed the comparison quickly, but then took time to deliberate confidence and difficulty ratings since response time was recorded only once all answers were given, and not when the subject selected “match” or “non-match”. Also, the self-confidence of the examiners in their abilities may have affected response time. Only a small component of the variability in response time was likely to be due to differences in attention or interest since such differences would presumably have led to greater variability in accuracy, which was not observed.

We fit a linear, mixed-effects model to normalized response time data for the training set following the same model selection steps as for the accuracy model described above. Due the variability in response time across experts, the random effect of expertID was retained in the model. The results of the regression are shown in Table 3. Three features, Core (L), Mean Block Contrast (K), and SD Block Contrast (L) were found to be predictive of response time using the same model selection procedure that was used for the analysis of predictors of comparison accuracy. The latter two predictors were also selected in models of accuracy (SD Block Contrast as part of an interaction term). Visibility of cores instead of deltas was selected as a predictor of response time. Interestingly, core visibility also appears as predictor when the model is fit to a testing set. Visibility of a core might make comparison of latents and known prints much easier: if the cores do not match then no further examination is required, so a comparison can be made quickly. Absence of a core could also make it difficult to orient the latent and known prints, since, as noted earlier, these features could act as landmarks for orienting two prints during comparison.

**Table 3:** Predictors for response time model.

Fixed Effects	Coefficient Estimates	Standard Error	*t*
Core (L)	−0.234	0.074	−3.149**
Intercept	0.226	0.085	2.646**
Mean Block Contrast (K)	−0.177	0.038	−4.647**
SD Block Contrast (L)	−0.105	0.038	2.748**
Random Effects	Variance		
printID	0.216		
expertID	0.226		

Note: ** *p*<0.01. Estimates are arranged by coefficient magnitude in descending order (see text). L – latent, K – known print, LxK – interaction.

Linear mixed-effects models were also fit separately for difficulty and confidence ratings. Like response time, there was a great deal of inter-subject variability for both measures. Variability in confidence and difficulty ratings may be due to differences in degree of expertise and self-confidence in the task. Variability in ratings may also be due to differences in interpretation of the rating task and therefore in response strategy. One expert, for example, responded with maximum confidence to all comparisons, saying to the experimenter that in real-world situations an expert would be 100% confident or rate a comparison as inconclusive.

Table 4 contains the coefficient estimates for the model of difficulty rating. As in the model of accuracy, Ridge Sum, Area Ratio, and Core (L) were selected as predictors. Similar to response time, difficulty was also negatively correlated with accuracy, so the regression coefficients have opposite sign to those in the accuracy model. In addition, visibility of Cores in the known print and the interaction of the Core terms were also selected. Delta (L) appears in this model as well as in the model of accuracy.

**Table 4:** Predictors for difficulty rating model.

Fixed Effects	Coefficient Estimates	Standard Error	t
Intercept	2.748	0.301	9.121***
Core (L x K)	−2.104	0.722	−2.913**
Core (L)	1.719	0.705	2.437**
Core (K)	0.935	0.324	2.883**
Delta (L)	−0.778	0.191	−4.082***
Ridge Sum	−0.207	0.079	−2.631**
Area Ratio	0.202	0.078	2.571**
Random Effects	Variance		
printID	1.076		
expertID	0.301		

Note: *** *p*<0.001, ** *p*<0.01. Estimates are arranged by coefficient magnitude in descending order (see text). L – latent, K – known print, LxK – interaction.

A similar model was fit for confidence ratings. The results are shown in Table 5. Identical predictors with comparable magnitudes were selected as for the difficulty rating model. The coefficients have opposite sign since high difficulty ratings correspond to low confidence ratings. Because difficulty and confidence are so strongly correlated (−0.91), it is not surprising that the exact same predictors are selected for in both models.

**Table 5:** Predictors for confidence rating model.

Fixed Effects	Coefficient Estimates	Standard Error	t
Intercept	5.248	0.247	21.255***
Core (L x K)	2.034	0.564	3.604***
Core (L)	−1.644	0.551	−2.983**
Core (K)	−0.920	0.253	−3.631***
Delta (L)	0.581	0.149	3.899***
Area Ratio	−0.162	0.062	−2.647**
Ridge Sum	0.155	0.062	2.517**
Random Effects	Variance		
printID	0.616		
expertID	0.488		

Note: *** p<0.001, ** p<0.01. Estimates are arranged by coefficient magnitude in descending order (see text). L – latent, K – known print, LxK – interaction.

## Discussion

We evaluated expert performance on a fingerprint matching task. Experts were highly accurate, committing relatively few errors despite limited access to resources and restricted viewing time. Using a number of potential predictors derived from image processing analyses, we were able to identify, using regression analyses, several image characteristics predictive of expert performance. Six features in particular were found to be important predictors of accuracy: Ridge Sum, Area Ratio, visibility of Deltas in the latent print, Mean Block Contrast of the known print, interaction between SD Block Contrast for latents and known prints, and the interaction between DEAI (deviation from expected average intensity) for the latents and known prints. Taken together, these features explain 64% of the variance in performance accuracy on a novel set of print pairs that were withheld from those used to train the model. A classifier derived from the full data set identified the pairs on which at least one expert made a mistake with 91% accuracy, and a similar model derived from 90% of the data classified novel pairs with 75% accuracy.

Many of the same image characteristics were also predictors of subjective difficulty ratings, confidence ratings, and response times. We also found that difficulty ratings, a subjective measure, were moderately correlated with accuracy and could improve the performance of the classifier on novel print pairs.

There are several interesting observations that can be made about the set of features that were found to be predictive of accuracy (Table 2). First, four of the six features were relational, in the sense that they were calculated based on information contained in both prints that make up a comparison pair. This is a desirable feature of the model since a particular print could arise in two separate comparisons (e.g., a latent print compared to a matching and a non-matching print). In real world scenarios, a single latent print may be compared to many known prints. In cases where one of the prints in the study was of very poor quality, then such relational features might not matter. For example, if Mean Block Contrast (K) is low (i.e., for a very washed out or very dark print), then a comparison would be difficult irrespective of some relational features such as Area Ratio. Conversely, if two prints do not share Level 1 pattern type, they will not make for a difficult comparison regardless of the quality and quantity of information in each. In general, however, error rates and difficulty seem likely to be primarily characteristics of print comparisons, rather than individual prints, as difficulty for actual non-match comparisons will be most acute when the prints share significant similarities, and difficulty for actual matches will be most acute when latent quality or quantity is limited or misleading. Results in our regression models support this idea.

Second, the features within the model correspond to many types of information content. Mean Block Contrast (K), SD Block Contrast (L x K), and DEAI (L x K) capture properties of the image itself (i.e., dark or light, uniform or not). Area Ratio and Delta (L) reflect large-scale or configural (Level I) characteristics of prints, and Ridge Sum relates to visibility of fine detail in the image such as Level II features (see Introduction). These outcomes fit broadly with the idea that fingerprint examiners access different kinds of information in making comparisons and that basic image characteristics determine the detectability of relevant features and patterns.

Third, the signs of the coefficients provide appealing interpretations. That high contrast and clarity of ridges are predictors of accuracy should not be surprising. The DEAI measure increases as the average pixel intensity approaches 127.5, the mean expected pixel intensity for an image that contains 50% white and 50% black pixels. We assumed that this proportion would correspond to greater clarity, since a mostly light or dark image could be difficult to analyze. The positive coefficient found for this measure in the accuracy model indicates that as the proportion of white to black pixels approaches 0.5 in the latent and known print, accuracy increases.

Visibility of deltas in the latent image also had a positive effect on accuracy perhaps because they provided orienting information, making it easier to match and compare feature locations on the latent and known print. Accuracy decreased as SD Block Contrast (L x K) and Area Ratio increased. When SD Block Contrast is high in both the latent and known print, accuracy is low. In general, high variability in Block Contrast picks up variable image quality across image regions (e.g., due to gaps or smudging in portions of a print). In smudged regions, pixels would be uniformly dark, while in clear regions pixel intensity would be more variable, leading to higher contrast measures in those areas. If an image were more uniform in pixel intensities, it would have lower variability in contrast across regions and therefore lower SD Block Contrast measures. Area Ratio had a large, negative coefficient. This at first seems counterintuitive; higher area ratios tend to correspond to larger areas of latent prints. One possible explanation might be that in the process of creating a database of print pairs with varying size latents that were not too difficult, small latents could perhaps have tended to be of higher quality while larger latents tended to be of lower quality. However, the evidence suggests that this explanation is not probable, because in fact, print area ratio and ridge reliability (perhaps the variable most directly corresponding to subjective image quality) were not correlated (*r* = 0.017, *p* = 0.415). Instead, we believe that latent prints with larger areas may be associated with more difficult comparisons because it is more difficult to identify distinctive regions of the image when the print is large. Since non-matching known prints were chosen by submitting the latents to an AFIS system, and selecting a non-match deemed to be the most similar of those available, the non-matches likely shared many features. If experts were only shown a small latent region, it might have been easier to compare that region to the corresponding region on the known print and quickly exclude mismatched pairs as compared to a larger latent image with more or larger, accidentally matching regions.

Difficulty ratings were used in two ways to add to the modeling results. We used difficulty rating itself as a predictor of accuracy. Difficulty ratings improved the fit of a model trained on all of the print pairs, but did not improve the predictive power of a model on a testing set of withheld prints. Classification performance, however, was improved. While ratings are not objective, there was nevertheless a moderate correlation between difficulty ratings and accuracy, suggesting that experts were reasonably aware of which comparisons were difficult. However, outside the experimental setting, it may be impractical to expect to be able to get a group of experts to provide ratings.

Difficulty ratings, confidence ratings, and response times were also evaluated as separate dependent measures. Because these measures correlated moderately with accuracy, we expected that similar features should be selected for when the same features were used to predict other dependent measures. Four of the six features that appeared in the accuracy model also appeared in the other models. A fifth feature, SD Block Contrast (K), which was included as part of an interaction term in the accuracy model appeared in the model of response time. Some features, such as visibility of cores, appeared in the other models but not in the model of accuracy. Cores and deltas are global features. Their presence or absence can be used as a quick measure of assessing difficulty. However, global features on their own are not sufficient to make a comparison. Accuracy, therefore, depends to a greater extent on image quality, relational information, and ridge information.

These results suggest that physical characteristics derived through image processing methods may be valuable in predicting expert difficulty and error rates for print pairs. Given that the present work is the first effort we know of to systematically predict errors from physical characteristics of print pairs, the predictive results are highly encouraging. Validation across larger data sets would be desirable for practical use of a predictive model such as the one derived here, further developments along these lines, along with continuing progress in characterizing the physical quality of prints (e.g., [9]) will likely prove to have practical value in quantifying the likely evidentiary value of expert assessments of fingerprint matches.

While these results on modeling print-pair difficulty are encouraging, there are also many differences between the paradigm used in the present study and the actual process of fingerprint comparison. In forensic settings, experts typically have unlimited evaluation time and access to image processing tools that were not available in the present study. In addition, examiners typically are not in a ‘forced-choice’ situation, and may decide that a real-world comparison is inconclusive rather than reaching a conclusion about match or non-match.

Despite these limitations, there are several important dimensions to these results. The experiment shows that even under constraints, experts were highly accurate. More than half of the print pairs had perfect accuracy, even in circumstances where the examiners' time was limited, their access to processing tools constrained, and in which they were not permitted to select the option of “inconclusive”. Relatively few studies have examined expert performance in fingerprint matching tasks, and this study adds to that body of research. Given the constraints imposed on examiners in this study, we would suspect that error rates in forensic laboratory settings could well be lower than those that we observed. For example, it is possible that had examiners had the option of choosing “inconclusive,” they might have elected to make that choice for some of the prints for which we saw the highest error rates. However, it is also possible that they might have selected “inconclusive” for prints for which they performed, individually or in aggregate, with a high degree of accuracy. Our data do not permit us to assess either of these possibilities; but this example illustrates why taking these data as offering an “error rate” would be both misleading and inappropriate.

Experiments in ecologically valid settings are difficult to conduct. Compared to this experiment, fingerprint examiners in actual practice encounter multiple factors that may improve accuracy (such as more time to conduct the comparisons, more access to tools, verification checks, etc.), as well as factors that can reduce accuracy (such as biasing influences from extraneous contextual case information, or pressures from investigators [19], [43]). Given the significant differences between our experimental conditions and ecologically-valid fingerprint identification, it is important to state clearly that the point of this experiment is *not* to measure error rates, and it would be a mistake to take these data as direct evidence of a specific error rate for the field [7]. Rather, we are interested in identifying the features that correlate with difficulty, in order to understand both what features of print pairs affect difficulty, and to begin to understand how error rate might *vary* with print difficulty.

This present study is therefore an important step in “unpacking” error rates, an endeavor that has great importance to forensic science and the legal system. The mere fact that some fingerprint comparisons are highly accurate whereas others are prone to error has a wide range of implications. First, it demonstrates that error rates are indeed a function of comparison difficulty (as well as other factors), and it is therefore very limited (and can even be misleading) to talk about an overall “error rate” for the field as a whole. In this study, more than half the prints were evaluated with perfect accuracy by examiners, while one print was misevaluated by 91 percent of those examiners evaluating it, and numerous others were misevaluated by several examiners. This distribution of errors strongly indicates that error rates do vary depending on the visual content of the specific comparisons. This experiment therefore provides strong evidence that print comparisons do vary in difficulty and that these variations also affect the likelihood of error.

Second, this study lays down a foundation for finding objective print characteristics that can quantify the difficulty of the comparison. The model we offer provides both evidence for what specific visual criteria seem to affect difficulty, as well as a model for combining these criteria to best predict accuracy. While further study regarding this model and its effectiveness in circumstances more ecologically valid than what was undertaken here is warranted before making definitive claims, this model illustrates the significant potential for creating objective measures of difficulty for print pairs.

Third, a more sophisticated understanding of the relationship between error rate and difficulty should be important for the courts in weighing fingerprint evidence (and the need for better information about the strengths and limitations of latent fingerprint comparison and other forensic techniques that has been highlighted by the NAS inquiry into forensic science [3]). Courts are instructed, when assessing expert evidence, to focus on the “task at hand”, and this research helps to show that fingerprint examination may vary in difficulty in ways that may be relevant to its evaluation as legal evidence [44], [45]. More nuanced assessments of fingerprint task difficulty might, for example, affect how a judge understands the admissibility of a specific fingerprint comparison, or what degree of certainty the expert will be allowed to express about a conclusion, or it might impact the weight or probative value given to a specific match conclusion by the fact-finder [46].

Fourth, the implication of these findings go beyond the courtroom; they also provide vital insights that can considerably enhance the work of forensic laboratories. For example, similar to medical triage, forensic laboratories may benefit from considering how the need for different procedures and checks can be made to fit the difficulty of the comparison (e.g., “the need for verification, and what sort of verification, may be highly dependent on the difficulty of the decision and the type and likelihood for a potential error. In cases with greater cognitive difficulty, when errors are more likely, more stringent verification procedures are needed; whereas more simple and straightforward prints may not require the same level and type of verification.” [47]).

Fifth, the understanding of what makes some comparisons more difficult than others has implications for the selection and training of fingerprint examiners. During examiner training, benchmarks and skill sets can be set as criteria to ensure candidates have the cognitive abilities needed to perform the tasks. Better understanding of which print comparisons are easier or more difficult can improve both training materials and the assessments of trainees. Training needs to have clear cognitive goals, and must use appropriate materials to develop and evaluate trainees' perceptual learning [12]. Understanding which comparisons are more error-prone can help improve both the design of training materials, to ensure a range of difficulty, and can also help evaluators assess performance in a more nuanced way.

## Supporting Information

Figure S1 **Screenshot of a sample trial from the experiment.** Examiners could use the keys in the windows to change position or zoom level. Responses were made by clicking on the buttons shown in gray. Once all responses were provided, a button appeared allowing the user to advance to the next trial.
(TIF)

Figure S2 **Global Feature GUI.** The interface used for counting deltas, marking the presence or absence of a core, and labeling the core type.
(TIF)
