# Supplementary figures and images for: Forensic Comparison and Matching of Fingerprints: Using Quantitative Image Measures for Estimating Error Rates through Understanding and Predicting Difficulty
Source: PLoS One. 2014 May 2;9(5):e94617. doi: 10.1371/journal.pone.0094617 (PMC4008377; doi:10.1371/journal.pone.0094617)

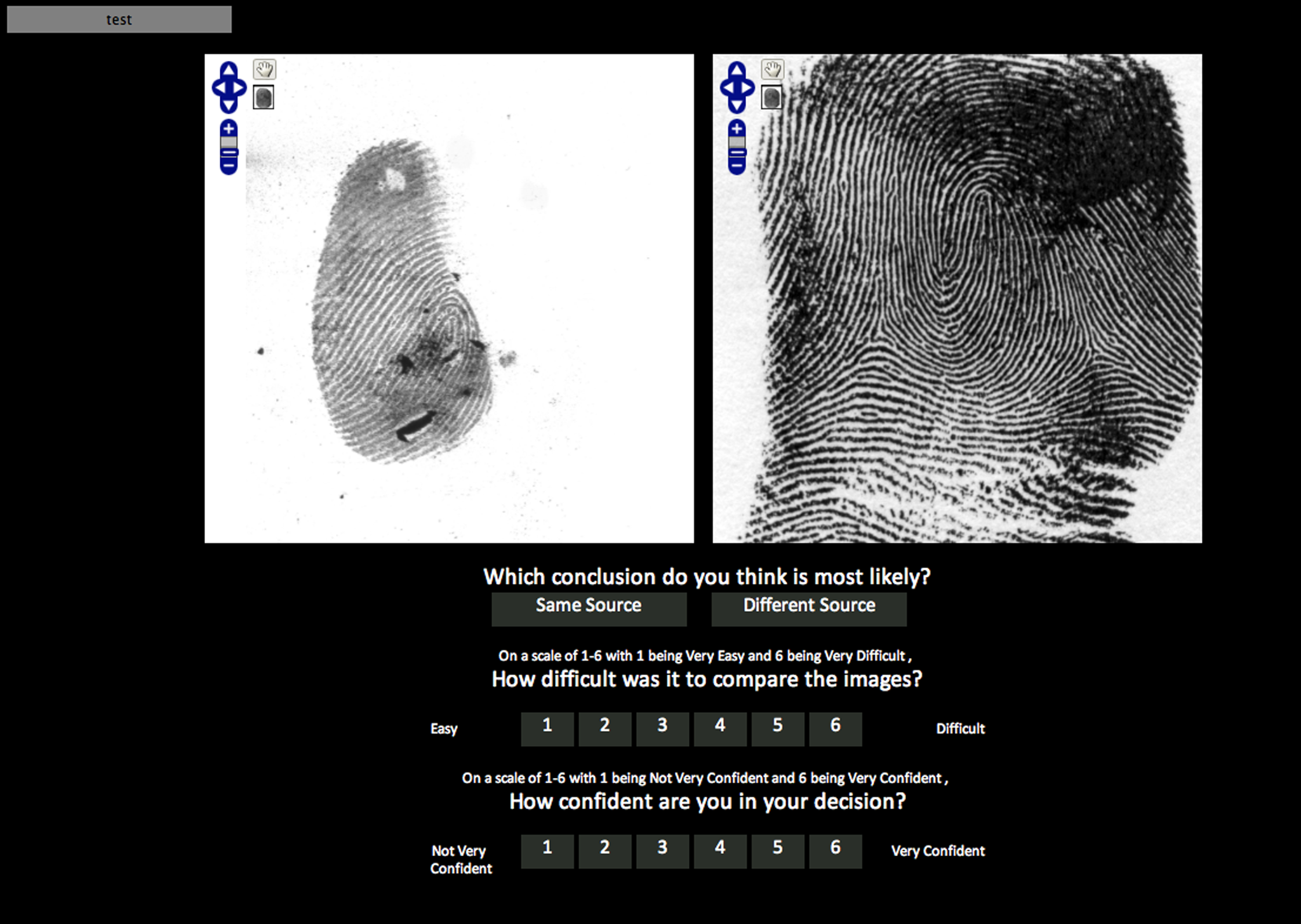

Supplement: Figure S1 — Screenshot of a sample trial from the experiment. Examiners could use the keys in the windows to change position or zoom level. Responses were made by clicking on the buttons shown in gray. Once all responses were provided, a button appeared allowing the user to advance to the next trial. (TIF) [file pone.0094617.s001.tif]

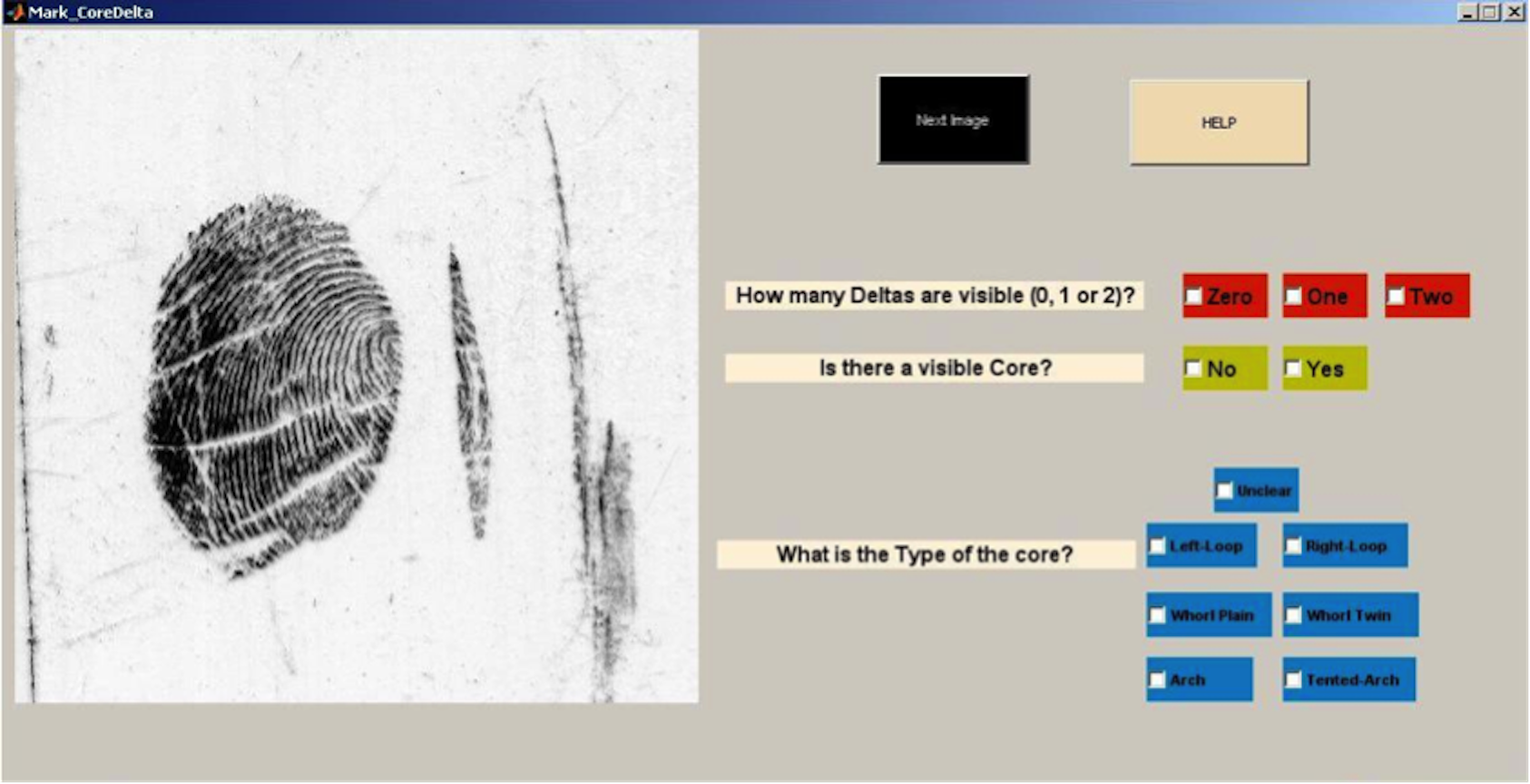

Supplement: Figure S2 — Global Feature GUI. The interface used for counting deltas, marking the presence or absence of a core, and labeling the core type. (TIF) [file pone.0094617.s002.tif]
